# Workplace violence against Chinese health professionals 2013–2021: A study of national criminal judgment documents

**DOI:** 10.3389/fpubh.2022.1030035

**Published:** 2022-10-19

**Authors:** Yu Xiao, Ting-ting Chen, Shao-yi Zhu, Ling Zong, Na Du, Chun-ya Li, Hao-fei Cheng, Qi Zhou, Li-shi Luo, Juan Jia

**Affiliations:** ^1^Psychosomatic Medical Center, The Fourth People's Hospital of Chengdu, Chengdu, China; ^2^Psychosomatic Medical Center, The Clinical Hospital of Chengdu Brain Science Institute, MOE Key Lab for Neuroinformation, University of Electronic Science and Technology of China, Chengdu, China; ^3^Nursing School, Chengdu University of Traditional Chinese Medicine, Chengdu, China; ^4^Department of Psychiatry, Shantou University Mental Health Center, Shantou, China; ^5^Department of Judicial Expertise, Zhongshan Third People's Hospital, Zhongshan, China; ^6^Institute (College) of Integrative Medicine, Dalian Medical University, Dalian, China

**Keywords:** health policy, health professionals, hospital violence, occupational safety, criminal judgment record, workplace violence

## Abstract

**Objectives:**

Patient-initiated hospital violence is a global problem which threatens the safety of health professionals and is indicative of doctor-patient tensions, impeding health system quality and access. The current study aimed to improve the understanding of medical workplace violence (WPV) in China, using authoritative and nationally representative judgment records, and to approach violence prevention strategies.

**Methods:**

All litigation records relating to violence against health professionals between 2013 and 2021 were extracted from the China Judgment Online System. Basic case information, victim characteristics, perpetrator characteristics and the nature of the violence were collated. The relationship between different treatment outcomes and violence was also explored.

**Results:**

Numbers of cases of hospital violence gradually increased from 2013 to a peak in 2016 before gradually decreasing in the following years. The most common perpetrators were patients' relatives (58.2%), followed by patients themselves (38.2%). Only 9 perpetrators had a confirmed history of mental illness and only two were intoxicated with alcohol. More than half of the cases (52.5%) occurred in rural areas and this percentage is even greater for primary health care institutions (71.4%) and secondary hospitals (73.5%). On a departmental level, the highest incidence of medical WPV was found in the emergency (18.9%), pediatrics (13.2%) and obstetrics (11.5%) departments. Violent behaviors, such as stalking, mass occupation of the ward and sharp instrument injury were significantly related to cases not involving patient death (*p* < 0.05). Disruptive behavior, such as hanging banners, blocking hospital passages, placing flower wreaths and burning paper money were significantly correlated with cases involving patient death (*p* < 0.01). The interval between a patient's death and the ensuing violence was short, happening on the same day in 54.8% of cases.

**Conclusions:**

A comprehensive overview of medical WPV in China is presented and may have utility for the formulation of prevention strategies.

## Introduction

Health care workplace violence (WPV) may be defined as physical or psychological incidents in which health professionals are abused, threatened or attacked at work (1). Threats to the safety, health or well-being of health workers may be explicit or implicit. Medical WPV is an occupational hazard faced by health professionals the world over and has caused concern both before and after the COVID-19 outbreak (2, 3). A 70–74% proportion of workplace attacks occur in medical facilities in the US (3) and 15% of health professionals responding to the 2019 UK annual survey reported experience of at least one physical violence incident from patients, relatives or other members of the public in the preceding 12 months (4). Most evidence of medical WPV has traditionally come from North America and Europe but has aroused attention as an increasing problem in developing countries. More than 30 South African hospitals reported serious safety incidents in just 5 months in a 2019 survey by the South African Medical Association (5) and 57% of medical ward staff in India experienced violence from patients or visitors (6). The prevalence of WPV against Chinese medical staff has steadily increased over the past decade to become a serious social problem (7). A national survey of Chinese healthcare workers gave the incidence of hospital violence as 65.8% (8).

A separate phenomenon of “Yi Nao” (medical disturbance or medical mobs) whereby patients express their dissatisfaction with an adverse outcome by protest and/or violence in hospitals has also been recorded (9, 10). Medical disturbance represents an organized and extreme form of WPV during which patients, relatives and even hired gangs attack health workers, disrupt the running of hospitals and damage hospital facilities in order to elicit monetary compensation. Yi Nao relating to patient death represents the lack of acceptance by the patient's family and may involve the holding of funerals, placing of flower wreaths, setting up of mourning halls and the burning of paper money on hospital premises with the disruption of normal service (11). More than 17,000 “medical troubles” took place in 2010, an increase of 7,000 compared with 5 years previously, according to Chinese health department statistics (11). WPV may result in physical and mental harm to health workers (8), including death, anxiety, depression and post-traumatic stress disorder (2, 12). It also translates into short-term and long-term economic costs to the workplace and reduces the quality of patient care (13). WPV has caused many Chinese health workers to leave the medical profession and has reduced the integrity of the doctor-patient relationship (14).

Previous studies on medical WPV in China have usually involved media searches, questionnaires or qualitative interviews (15–17). The shortcomings of such studies include the cross-sectional nature and the failure to follow up on violence resolution. A better understanding of the consequences of violence would aid policymakers in the formulation of prevention strategies. A second disadvantage is that media reports often tend to involve eye-catching cases (18) and under-report the total number of incidents, failing to give the whole picture. The third disadvantage is a focus on hospitals in a certain province and the failure to be nationally representative (19).

Litigation records relating to medical malpractice have been used as a comprehensive and reliable data source in developed countries (20) but such an approach is of limited utility in developing countries. The current study used authoritative national judgment documents to evaluate medical WPV and a discussion of the formulation of violence prevention strategies in China is presented. To the best of our knowledge, the current study will be the most up-to-date exploration of medical WPV in China.

## Methods

### Database

Data was downloaded from the China Judgment Online System (CJOS) which is managed by the Supreme People's Court of China and represents the most reliable and comprehensive national database of judgment documents (21). Case judgments of all courts in 31 Chinese provinces may be accessed via CJOS. Judgment documents have been routinely published within 7 days of sentencing since 2013 and the online system accommodated more than 100 million judgment documents by the end of 2021.

### Search strategy and exclusion criteria

All criminal judgment documents reported the date of occurrence of the crime and the date of sentencing by the court. All cases of medical WPV were included from the date of CJOS inception, January 1st, 2013, to the latest date for complete annual records, December 31st, 2021.

Three key phrases were initially used to screen judgment documents: “medical institutions”, “criminal cases” and “health care workers”. An advanced search was conducted using “criminal cases” and the other two phrases were used as keywords for the whole text. The following more specific terms, including “criminal cases”, “hospital”, “clinic”, “health room”, “health center”, “maternal and child health hospital”, “community health center”, “doctor”, “physician”, “nurse”, “radiographer”, “pharmacist”, “technician”, “laboratorian” and “health worker” were used to identify further cases involving medical WPV. The preliminary search returned 54,618 criminal cases.

**Figure 1:**
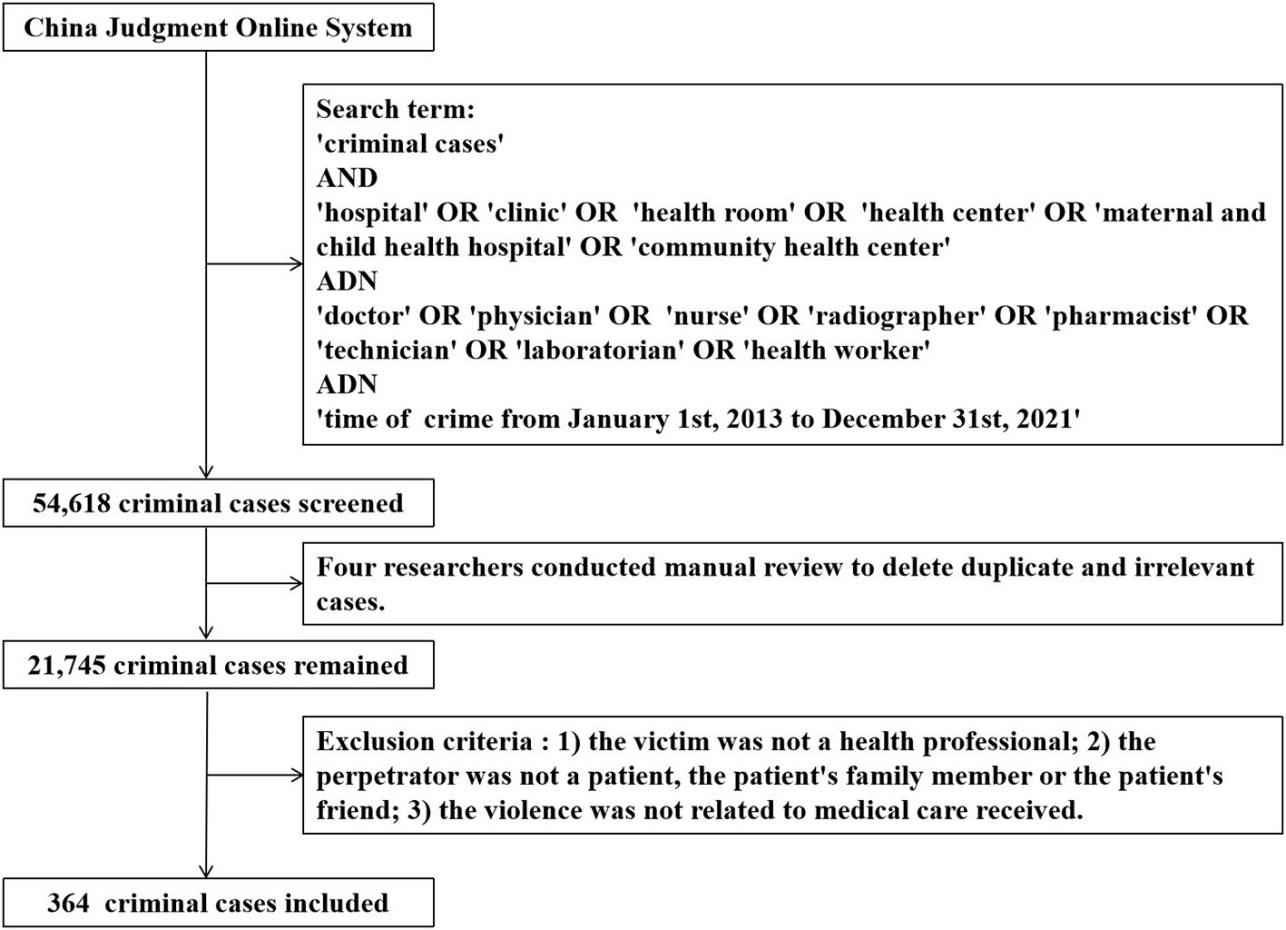
Flow chart of judgment document extraction.

All judgment documents were manually screened by four researchers and irrelevant cases in which there was no violence against health professionals deleted. A total of 21,745 cases were retained. In cases where more than one hearing was held, such as an initial hearing followed by an appellate court hearing, only the latter was included to avoid duplication. Exclusion criteria were as follows: 1) the victim was not a health professional; 2) the perpetrator was not the patient, a member of the patient's family or the patient's friend; 3) the violence was not related to the medical care received. A final total of 364 non-repetitive cases were included. Figure 1 shows a flow chart detailing the process of document extraction.

### Variable coding and statistical analysis

Judgment files were encoded into four groups of numerical and string variables in standard data format, as follows: (1) Basic case information, including court level, crime date and document number; (2) Characteristics of health professionals, including work department and occupation (e.g., doctor), type and location of health care facilities and whether the death of a health professional was involved; (3) Characteristics of perpetrators, including gender, age, occupation, educational level, relationship with patient, assumption of criminal responsibility, history of mental illness or alcohol intoxication and whether medical malpractice technical authentication had been conducted; (4) Characteristics of the violence, including type, whether patient death was involved and the interval between patient death and the violence.

In cases involving more than one defendant, the first defendant's information was taken to represent that of all defendants. Original data was input simultaneously by two researchers to establish inter-rater reliability. Four researchers completed independent reviews of the variable codes to account for potential variation among coders. Discrepancies in variable coding were resolved by team discussion to achieve consensus.

Researchers produced a descriptive statistical analysis of the characteristics of medical WPV and cases involving patient deaths were investigated as representing the most serious adverse consequence. The time interval between the occurrence of the adverse outcome, such as patient death, and violence was considered and the possibility that a death deemed unacceptable by the patient's family might precipitate the seeking of revenge. Cases involving patient death were compared with those where no death occurred and the correlation between violence and the patient's dead/alive status assessed by Fisher's exact test with IBM SPSS 22.0. Health facilities were categorized according to the Chinese Health Statistics Yearbook 2021 (22). All descriptive statistical analyses and testing of hypotheses were conducted by Microsoft Excel 2019 and IBM SPSS 22.0 with a value of *p* < 0.05 considered to indicate statistical significance.

## Results

**Figure 2:**
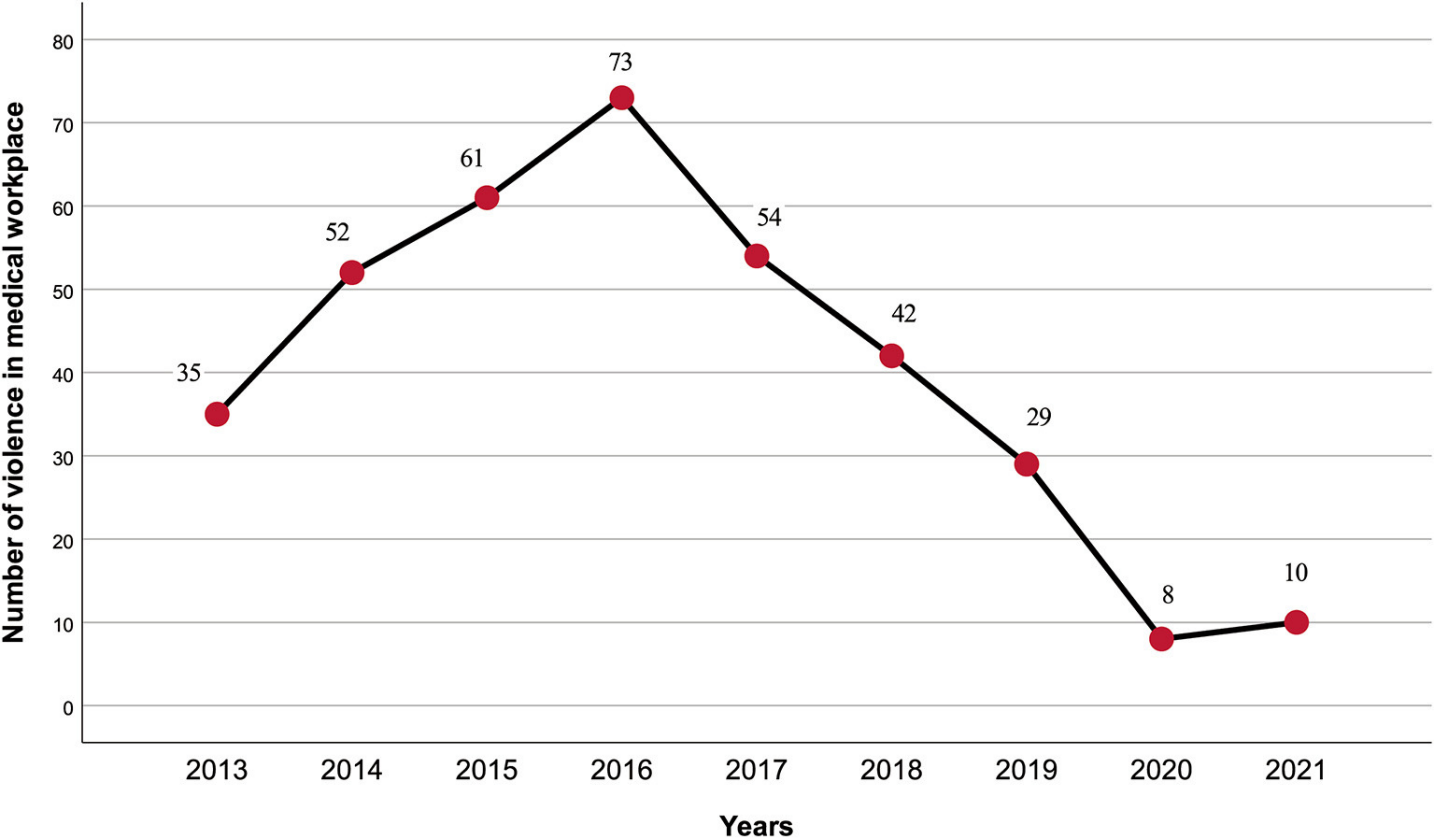
The frequency of medical workplace violence between 2013 and 2021.

**Figure 3:**
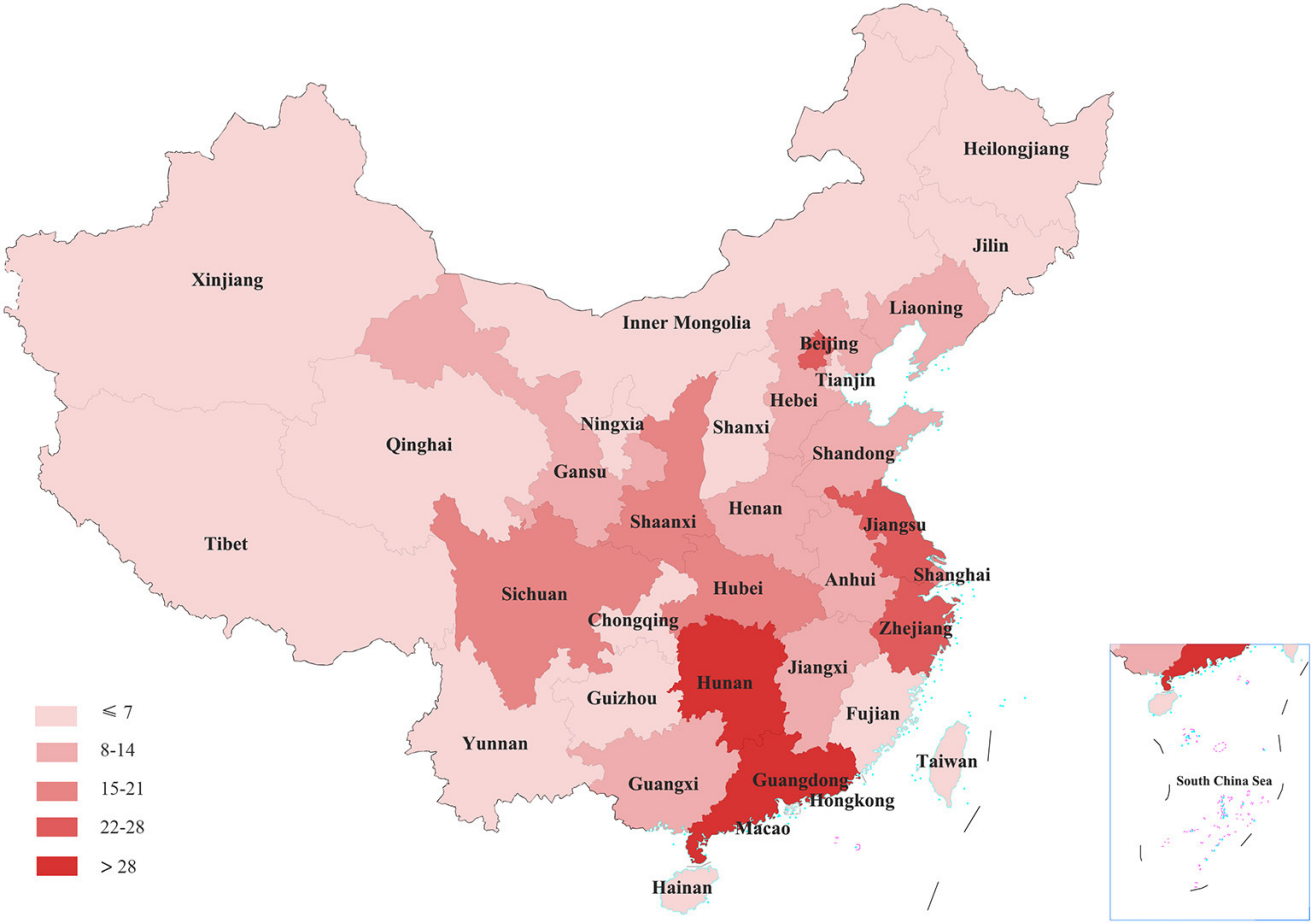
The distribution of medical workplace violence cases in different provinces of China.

A total of 364 cases related to medical WPV were tried by Chinese courts between 2013 and 2021. A trend of increasing hospital violence, followed by a decrease can be seen over the last 10 years (Figure 2). The incidence of WPV cases showed a year-on-year increase from 2013 (*n* = 35), reaching a peak in 2016 (*n* = 73) and gradually decreasing in the following years. By the end of the timeframe of interest, the rate had dropped to the lowest level (*n* = 10), less than one-third of the rate in 2013. The distribution of medical WPV cases by province can be seen in Figure 3, in which greater numbers of cases are represented by darker colors. The following five provinces/municipalities had the most cases of WPV: Guangdong, 53 (14.6%); Hunan, 29 (8.0%); Beijing, 27 (7.4%); Jiangsu, 26 (7.1%) and Zhejiang, 23 (6.3%). The majority of medical WPV cases were concentrated in Eastern regions with the highest numbers in Guangdong (Eastern) and Hunan (central) provinces and no cases were reported from the Western provinces of Qinghai (*n* = 0) or Tibet (*n* = 0).

### Information about the perpetrators

Demographic characteristics of perpetrators are shown in Table 1. Most perpetrators were male (*n* = 239; 81.8%), aged between 18 and 47 (68.8%), had not finished high school education (66.7%) and 80.9% were farmers or unemployed. The most common perpetrators were members of patients' families (58.2%), followed by patients themselves (38.2%). The vast majority of perpetrators bore full criminal responsibility (*n* = 353; 96.9%) with only 9 (2.5%) having a history of mental illness and only two being intoxicated with alcohol. No records of intoxication with illegal drugs were found.

**Table 1:** Demographic characteristics of perpetrators.

**Category**	***n***	**%**
**Gender**		
Male	239	65.7
Female	53	14.6
Not reported	72	19.7
**Age (years)**		
< 18	2	0.5
18~27	15	4.1
28~37	80	22.0
38~47	44	12.1
48~57	53	14.6
58~67	7	1.9
>67	1	0.3
Not reported	162	44.5
**Occupation**		
Farmer	137	37.6
Unemployed	58	15.9
Worker	33	9.1
Self-employed	11	3.1
Student	2	0.6
Not reported	123	33.7
**Relationship with patients**		
The patient's family	212	58.2
Patient	139	38.2
The patient's friend	13	3.6
**Education level**		
Illiterate	17	4.7
Primary school	44	12.1
Junior high school	53	14.5
Senior high school	49	13.5
Above college degree	8	2.2
Not reported	193	53.0
**History of mental illness**		
Yes	9	2.5
No	355	97.5
**Intoxicated with alcohol at perpetration**		
Yes	2	0.5
No	362	99.5
**Conducted medical malpractice technical authentication**		
Yes	33	9.1
No	331	90.9
**Capacity for responsibility**		
Full criminal responsibility	353	96.9
Diminished criminal responsibility	11	3.1
Criminal incapacity	0	0.0

### Information about the victims

The occupational categories of victims included doctors, nurses, laboratory technicians or radiographers and others (e.g., security personnel). The vast majority of cases concerned doctors (59.9%), followed by nurses (26.1%). Secondary hospitals were the sites of 24.2% of cases, primary health care institutions of 13.5% and tertiary hospitals of 12.9% (Table 2). Violent incidents also took place in other types of hospitals, such as Chinese medicine hospitals (9.3%), specialist hospitals (8.2%) and private hospitals (6.0%). More than half of the cases (52.5%) occurred in rural areas with this percentage being even greater where primary health care institutions (71.4%) and secondary hospitals (73.5%) were involved. Of hospital-based WPV, teaching hospitals accounted for 74.7% (*n* = 272) of incidents and non-teaching hospitals for 20.6%. Cases showed a wide distribution across 10 clinical departments but the three with the highest incidences were the emergency (18.9%), pediatrics (13.2%) and obstetrics (11.5%) departments. Seven health care workers were murdered.

**Table 2:** Information about the victims.

**Category**	***n***	**%**
**Profession**		
Doctor	218	59.9
Nurse	95	26.1
Laboratory technician or radiographer	7	1.9
Others	40	11.0
Not reported	4	1.1
**Department**		
Emergency department	69	18.9
Pediatrics	48	13.2
Obstetrics	42	11.5
Internal medicine	26	7.1
Orthopedics	15	4.1
Surgery	13	3.6
Reproductive health	11	3.0
Oncology	8	2.2
Ophthalmology	5	1.4
Otolaryngology	5	1.4
Not reported	122	33.6
**Location**		
Urban	160	44.0
Rural	191	52.5
Not reported	15	3.5
**Health care facilities**		
Primary health care facilities	49	13.5
Secondary hospital	88	24.2
Tertiary hospital	47	12.9
Chinese medicine hospital	34	9.3
Specialized hospital	30	8.2
Private hospital	22	6.0
Private clinic	19	5.2
Other hospital	58	15.9
Not reported	17	4.8
**Teaching hospital**		
Yes	272	74.7
No	75	20.6
Not reported	17	4.7

### Information about violence

#### Overview of various types of violence in criminal cases

The various types of violence detailed by judgment records are summarized in Table 3 and are divided into 3 categories: psychological violence, physical violence and medical disturbance (“Yi Nao”). Verbal abuse amounting to psychological violence occurred in 28.3% of cases, and 18.1% involved threats. Physical violence ranged in severity from pushing to bombing. Beating accounted for the highest proportion (43.1%), followed by pushing (30.5%) and 14.0% involved injury by a sharp instrument, such as a dagger. The four most common modes of “Yi Nao” included hanging banners (36.3%), destroying hospital equipment (34.3%), blocking hospital passages (31.0%) and burning mock paper money (26.4%).

**Table 3:** Types of violence in the judgement records.

**Violence**	**Total**	**%**	**Patient died**	**%**	**Patient alive**	**%**	**Fisher's exact test**
Overall	364		229		135		
**Psychological violence**							
Verbal abuse	103	28.3	73	31.9	30	22.2	0.161
Threatening	66	18.1	39	17.0	27	20.0	0.582
Stalking	12	3.3	0	0.0	12	8.9	0.000*
Occupy hospital ward	11	3.0	3	1.3	8	5.9	0.025*
Besieging	26	7.1	21	9.2	5	3.7	0.089
Extortion	3	0.8	0	0.0	3	2.2	0.052
**Physical violence**							
Sharp instrument injury	39	10.7	7	3.1	32	23.7	0.000*
Blunt instrument injury	51	14.0	27	11.8	24	17.7	0.218
Beating	157	43.1	93	40.6	64	47.4	0.434
Biting	37	10.2	29	12.7	8	5.9	0.072
Pushing	111	30.5	81	35.4	30	22.2	0.054
Electric shock	2	0.5	0	0.0	2	1.5	0.139
Water gun	15	4.1	7	3.1	8	5.9	0.277
Explosion	12	3.3	7	3.1	5	3.7	0.768
**Medical disturbance**							
Placing flower wreath	45	12.4	45	19.7	0	0.0	0.000*
Burning paper money	96	26.4	96	41.9	0	0.0	0.000*
Burning incense	15	4.1	15	6.6	0	0.0	0.002*
Letting off firecracker	30	8.2	28	12.2	2	1.5	0.000*
Placing the photograph of the dead	13	3.6	13	5.7	0	0.0	0.005*
Placing the corpse in the hospital	43	11.8	43	18.8	0	0.0	0.000*
Playing funeral music	17	4.7	17	7.4	0	0.0	0.001*
Mourning	31	8.5	31	13.5	0	0.0	0.000*
Hanging banner	132	36.3	117	51.1	15	11.1	0.000*
Shouting using amplifier	11	3.0	10	4.4	1	0.7	0.063
Posting online	14	3.8	10	4.4	4	3.0	0.586
Blocking hospital passages	113	31.0	88	38.4	25	18.5	0.003*
Throwing feces	6	1.6	5	2.2	1	0.7	0.421
Blocking the checkout counter	39	10.7	30	13.1	9	6.7	0.113
Destroying hospital equipment	125	34.3	81	35.4	44	32.6	0.747
Throwing stone	11	3.0	9	3.9	2	1.5	0.341

Percentage: the proportion of cases reporting certain kind of violence in each category. Factors significant at 0.05 level were marked with “*”.

#### The relationship between violence and treatment outcome of patients (dead or alive)

Records of WPV cases showed that 229 relevant patients died and 135 patients did not. Forms of violence, such as stalking (*p* < 0.01), refusing to leave wards (*p* < 0.05) and blunt instrument injury (*p* < 0.01), showed a significant association with cases that did not involve patient death (Table 3). By contrast, hanging banners, blocking hospital passages, placing flower wreaths, burning paper money and placing the corpse in the hospital were significantly correlated with cases involving patient death (*p* < 0.01). Verbal abuse and blunt instrument injury showed no relationship with patient death.

#### Other features of medical WPV

Violence followed patient death with a much shorter time interval than for cases not involving the death of patients. Among the cases of violence following patient death, 54.8% occurred on the same day as the dispute happened, and 22.6% occurred within 1 week. Only in 21 cases was technical authentication of medical malpractice, requiring a third-party medical association to assess whether the adverse results were due to negligence, implemented. The court determined that 14 cases were due to medical malpractice. Four people were sentenced to death for murder as a result of medical WPV although most (82.4%) have been sentenced to fixed-term imprisonment. It is noteworthy that the police were attacked by angry patients or their families while maintaining order in 68 cases (18.7%).

## Discussion

The purpose of the present study was to employ the underutilized data source of public litigation records to analyze medical WPV. Litigation records are a valuable asset for research into health policy in developing countries and have many advantages. Firstly, judgment documents are more nationally representative than previous surveys conducted in some Chinese provinces. Secondly, previous investigations into hospital violence have been conducted from the perspective of health professionals and have neglected to focus on patients and their families (perpetrators). The judgment record gives offender characteristics that previous studies have failed to capture, such as the assumption of criminal responsibility and whether a medical malpractice suit has been brought. Thirdly, our research includes medical disturbance or “Yi Nao” as an alternative form of violence which has rarely been mentioned in international literature. Collation of judgment documents has allowed us to contribute information on perpetrators to the existing literature which would otherwise have been excluded from a cross-sectional study or media search. Pre-existing challenges faced by Chinese medical institutions in dealing with WPV have been exposed in such a way as to facilitate the design of prevention strategies.

WPV was found to rise sharply in 2013 before declining after 2016, perhaps due to the revision of the Criminal Law of the People's Republic of China in 2015 (23). A legal amendment allowed courts to impose more than 3 years' imprisonment on criminals who gather people to disturb hospital order, causing heavy losses (23). The availability of severe legal punishments has previously been shown to act as a deterrent, decreasing the cost of crime and reducing the incidence of hospital violence (18). However, the number of relevant cases in 2021 was slightly higher than that in 2020 and the COVID-19 epidemic may be partly responsible. A recent systematic review demonstrated an increase in the incidence of WPV during the COVID-19 epidemic with prevalence rates ranging between 5.8 and 44.4% for physical and 9.6–97.6% for verbal violence (2). Thus, the heavy workload, stressful work conditions and inadequate human and material healthcare resources that accompanied the pandemic may have increased the vulnerability of health professionals to WPV. The spread of SARS-CoV-2 around the world continues and governments of the USA, China, India and Algeria have introduced emergency laws to protect health professionals (24). A regional variation in medical WPV was revealed with incidents more likely to take place in medical institutions of the Eastern and central regions between 2013 and 2021 than those located in the Western provinces. Population size and the number of institutional visits have been identified as contributory factors (23) but health care policy and the medical system, including healthcare financing and regulation, should also be considered.

Doctors were found to be more likely to experience WPV than nurses. Previous studies have shown that nurses receive more verbal violence than doctors (25) and doctors are more often victims of physical violence in the workplace (26). These results indicate that the most serious incidents of WPV may be caused by major problems, such as diagnosis and treatment decisions, which are usually taken by doctors. WPV was found to occur widely in health care facilities at all levels, with tertiary hospitals accounting for only 12.9%, consistent with previous conclusions (27). More than half of the medical WPV incidents occurred in rural primary health care institutions or secondary hospitals. By contrast, Ma et al. (23) used media reports from 2004 to 2018 and concluded that serious WPV was largely restricted to cities (90.2%), usually occurring in tertiary hospitals (67.9%). There are two possible explanations for these differences. Tertiary hospitals are the highest-ranking hospitals in China, usually with more qualified doctors and a higher quality of care whereas lower-level hospitals tend to provide lower-quality care, leading to serious patient dissatisfaction and more frequent WPV against medical staff (28). Higher profile tertiary hospitals are usually located in big cities and violent cases are more likely to be reported by the media (18). However, the current findings suggest that medical WPV in low-level facilities and rural areas may have been underestimated. The graded diagnosis and treatment system currently being introduced in China (29) may funnel more patients into primary medical institutions, necessitating further research in the future.

It is noteworthy that many more WPV cases occurred in teaching hospitals than in non-teaching hospitals. Many doctors in Chinese teaching hospitals undertake scientific research and teaching, reducing the time available for clinical duties (28). Routine inpatient service is often performed by postgraduate students, resident trainees and further educational doctors who have less experience in interacting with patients and fewer medical skills which may increase the possibility of involvement in medical disputes (30). Staff in the emergency, obstetrics and pediatrics departments were the most vulnerable to WPV, a finding consistent with those of other studies (23, 31). However, a specific specialization was not reported for many cases meaning that this result should be interpreted with caution.

Studies from abroad have shown that drug abuse is a common trigger of violence and the impulsiveness of drunken patients or companions also poses a threat to the safety of doctors and nurses (32). Chinese health professionals are often warned to be wary of “high-risk” tourists, who may be drunk or have mental disorders (3). However, the current results showed that very few perpetrators suffered from mental illness nor were alcohol or drugs precipitating factors. Intoxication and mental illness do not seem to be the main characteristics of perpetrators (27). Perpetrators were often relatives of patients rather than patients themselves, a phenomenon that has been reported across different time periods, including the COVID-19 pandemic, and different cultures (2, 33). Family members may suffer psychological stress and have to deal with complicated medical procedures, expenses and other issues, generating more quarrels (31). Perpetrators were found to be generally poorly educated and many were farmers or unemployed, consistent with previous studies (7, 31). The level of medical knowledge of patients or their family members with the above characteristics is likely to be low and may be accompanied by unreasonable treatment expectations. If expectations are not met, violence may become a way to vent dissatisfaction (34). Health policymakers might consider promoting medical literacy education to enhance the public's understanding of medicine (35).

Different treatment outcomes were found to be related to different types of violence. Where patient death was not involved, stalking and blunt instrument injury were more common forms of violence. Where patient death occurred, medical disturbances, such as the burning of paper money, hanging of banners and blocking of hospital passages, were more likely to follow. Medical disturbance is a phenomenon unique to China and constitutes a form of hospital violence (17, 20), being categorized as a public order offense in criminal law since 2015 (11). However, medical disturbance is on the increase as a result of hospital managers fearing damage to the reputation and performance of hospitals from adverse treatment outcomes and the consequent payment of monetary compensation to resolve conflicts quickly (11). This suggests that tedious medical malpractice suits may not be the first choice for hospitals and patients. In addition, verbal abuse was the most common psychological violence experienced by health professionals and beating was the most common physical violence. Preservation of the mental health of those in high-pressure working environments and self-defense are challenges that deserve attention (2).

More than half of violent incidents consequent on a patient's death occurred within 1 day and more than 76% within 1 week. Death was clearly the trigger of violence (17, 36), reflecting the supremacy of life in Chinese culture and the difficulty that family members often have in accepting the sudden death of a patient. Perpetrators desired either compensation or revenge to assuage their dissatisfaction (20). A similar phenomenon has been reported in India (37) with disgruntled patients preferring violent revenge rather than a legal resolution. Chinese medical education tends to lack instruction in human interaction or in communication skills (14). Instruction in notifying the family of a patient's death and explaining the circumstances would be beneficial. Police officers were sometimes attacked while maintaining order and may also benefit from instruction in self-protection when dealing with hospital violence and in preventing the spread of violence.

Two recent policy responses to medical WPV have been put forward. In 2013, China's Ministry of Health issued the “Guideline on Strengthening Security Systems in Hospitals” (38) which recommended the allocation of one security guard for every 20 beds. Since 2020, Beijing has strengthened hospital security checks to prevent admission of visitors carrying knives (39). However, some scholars have condemned these policies as “treating the symptom but not the disease” since the focus is on strengthening security, rather than correcting the underlying social factors and the structure of the health system (9).

The current study fills knowledge gaps left by previous research based on questionnaires, media reports or interviews and informs China's prevention strategies. We find that merely tightening security in hospitals is insufficient to address medical WPV, a result which has been confirmed by other studies (2, 11, 18). Better strategies to solve cultural and organizational factors in the workplace and better cooperation between occupational and public health stakeholders are necessary to improve the occupational safety of health professionals (2). Multi-department cooperation, strengthening of doctor-patient communication, improving the legal system of medical malpractice authentication and establishing a complete hospital violence crisis management system are suggested to be appropriate future directions.

## Limitations

We acknowledge some limitations to the current study. First, only pre-existing data is included since information of ongoing cases was not available. Second, only adjudicated litigation cases were included and those settled or mediated out of court were not. Out-of-court cases tend to be less serious, so we concentrated on the more serious cases. Third, demographic information on perpetrators was incomplete in some cases due to inconsistent record keeping. Only available data could be analyzed and results should be interpreted on that cautionary basis. Fourth, a single data source was used and this could be seen as a limitation. However, the CJOS is a reliable and underutilized data source in China. Fifth, the focus on criminal judgment records means that emphasis was put on physical violence and medical disputes. Psychological violence against health workers may have long-term impact and merits more research in the future. Finally, under the unique cultural background of China, different forms of medical disturbance often influence each other. Therefore, the relationship between these violence variables and WPV is very complex and needs further study in the future.

## Conclusion

Characteristics of violence against health professionals in China were collated from litigation records and the formulation of violence prevention policies is discussed. Doctors were more vulnerable to WPV than nurses and violence occurred in all types of health institutions in both urban and rural areas. Very few perpetrators suffered from mental illness or were intoxicated with alcohol or drugs nor was medical malpractice authentication often involved. In addition, perpetrators were often the patients' relatives not the patients themselves. Different forms of violence followed different treatment results and patient death was found to be a significant trigger. It is recommended that China's current policies on violence prevention should extend beyond the tightening of hospital security. Coordination of structural, organizational and individual participatory interventions is required to ameliorate the situation.

## Data availability statement

The raw data supporting the conclusions of this article will be made available by the authors, without undue reservation.

## Author contributions

Data curation: YX, ND, LZ, and C-yL. Investigation: H-fC, QZ, L-sL, and JJ. Writing—original draft: YX. Writing—review and editing: T-tC and S-yZ. All authors contributed to the article and Approved the submitted version.

## Conflict of interest

The authors declare that the research was conducted in the absence of any commercial or financial relationships that could be construed as a potential conflict of interest.

## Publisher's note

All claims expressed in this article are solely those of the authors and do not necessarily represent those of their affiliated organizations, or those of the publisher, the editors and the reviewers. Any product that may be evaluated in this article, or claim that may be made by its manufacturer, is not guaranteed or endorsed by the publisher.

## Abbreviations

WPV: Workplace Violence
CJOS: China Judgment Online System.
